# Metrology of Supraspinatus Tendon Thickness in Swimmers with Tendinopathy Using Ultrasound Imaging: An Intra- and Inter-Rater Reliability Study

**DOI:** 10.3390/jcm13133822

**Published:** 2024-06-29

**Authors:** Sebastian Klich, Magdalena Podczarska-Głowacka, Juan Antonio Valera-Calero, José Luis Arias-Buría, Cesar Fernández-de-Las-Peñas

**Affiliations:** 1 Department of Paralympic Sport, Wrocław University of Health and Sport Sciences, 51-612 Wrocław, Poland; 2 Department of Basic Physiotherapy, Gdansk University of Physical Education and Sport, 80-336 Gdańsk, Poland; magdalena.podczarska-glowacka@awf.gda.pl; 3 Department of Radiology, Rehabilitation and Physiotherapy, Faculty of Nursery, Physiotherapy and Podiatry, Complutense University of Madrid, 28040 Madrid, Spain; juavaler@ucm.es; 4 Grupo InPhysio, Instituto de Investigación Sanitaria del Hospital Clínico San Carlos (IdISSC), 28040 Madrid, Spain; 5 Department of Physical Therapy, Occupational Therapy, Physical Medicine and Rehabilitation, Universidad Rey Juan Carlos, 28922 Madrid, Spain; joseluis.arias@urjc.es (J.L.A.-B.); cesar.fernandez@urjc.es (C.F.-d.-L.-P.)

**Keywords:** B-mode, ultrasound, rotator cuff, tendinopathy, overhead movements, swimmers

## Abstract

**Objective:** This study aimed to assess the relative and absolute intra- and inter-rater reliability of supraspinatus tendon (SST) thickness. **Materials:** Thirty adolescent swimmers with supraspinatus (SS) tendinopathy (*n* = 15) and a control-matched group (*n* = 15) were evaluated. Tendon thickness was measured according to four different measure procedures, i.e., (1) at 15 mm, (2) at 10, 20, and 30 mm, (3) at 10, 15, and 20 mm, and (4) 5 and 10 mm lateral to the most hyperechogenic reference point of the biceps tendon. Each examiner took two US images for the test measurements with a 10 min rest period. After 30 min, the subjects underwent retest measurements that were also repeated 1 week later. **Results:** SST thickness was greater in swimmers with SS tendinopathy compared with the matched control group for each procedure and rater (*p* < 0.001). Intra- and inter-rater reliability was good to excellent (ICC2.3: 0.78–0.98 and 0.83–0.97, respectively) in both groups. The lowest intra- and inter-rater reliability was found in procedures no. 2 and 4 (ICC2.3: 0.78 and 0.83). However, procedure no. 3 was the most reliable with the lowest error rate (ICC2.3: 0.92–0.97; SEM: 0.05–0.10 mm; MDC: 0.14–0.28 mm). **Conclusions:** The study confirmed the diagnostic value of ultrasound in SS tendinopathy. A multiple-reference-point procedure including a simple methodology (10, 15, and 20 mm from biceps tendon), was defined as the most reliable, expressed by the highest intra- and inter-rater ICCs.

## 1. Introduction

Ultrasound imaging (US) is a proven imaging method able to examine soft tissue structures, including tendons, their vascularization, and thickness. Compared to other imaging methods, such as MRI and CT, the US appears to be advantageous due to its non-invasiveness, radiation-free nature, and capability for real-time assessment [1]. Furthermore, studies have demonstrated the benefits of US in tendon measurements, attributed to its ability to provide high image resolution [2].

One of the common musculoskeletal dysfunctions in swimmers is shoulder impingement syndrome. Swimming training involves repetitive overhead movements. In all main swimming strokes (freestyle, backstroke, breaststroke, and butterfly), the swimmer uses large moment arm forces to reach forward to drag the water. When training is intense, all of these factors can contribute to shoulder dysfunction and pain. Supraspinatus (SS) tendinopathy is one of the causes of “swimmer’s shoulder” [3]. The supraspinatus is the primary responsible rotator cuff muscle for securing the humeral head into the glenoid and its tendon is susceptible to tendinopathy in swimming. Research indicates a problem in the acromiohumeral distance and a reduction in the subacromial space as contributing factors to subacromial impingement syndrome [4,5,6].

Previous studies have assessed the thickness of the supraspinatus tendon (SST) in healthy swimmers, which ranged from 5.41 mm to 7.94 mm [7,8,9,10,11], while Porter et al. [12] reported an SST thickness of 7.51 mm in swimmers with shoulder pain. The intra- and inter-rater reliability was investigated by using various statistics approaches, including the intra-class correlation coefficient (ICC) [1,6,10,13,14], standard error of measurement (SEM) [1,10], and minimal detectable change (MDC) [1,6,7,10]. These studies showed their intra- and inter-rater reliability were excellent (>0.92), with a SEM between 0.05 mm and 0.33 mm, and an MDC ranging between 0.40 mm and 0.91 mm. Some studies have evaluated SST thickness at a single SST location, i.e., 10 mm [11] or 15 mm [5] measured lateral to the long biceps tendon. Kretić et al. [14] measured SST thickness in the thickest or middle portion of the tendon. Recently, some procedures have investigated SST thickness at multiple locations [4,6,13]. Leong et al. [13] evaluated the SST thickness at a distance of 10-20-30 mm, Michener et al. [6] at 10–15–20 mm, while McCreesh et al. [4] at 5 mm and 10 mm lateral to the most hyperechogenic reference point of the biceps tendon. The current body of literature supports the assessment of SST thickness at several locations, even if its relative value and absolute reliability have not been reported yet.

Although ultrasound measurement is one of the most commonly used methods to detect rotator cuff dysfunction, there is still not enough published data regarding its reliability. According to previous studies, there is still a need for further investigation in rotator cuff ultrasound imaging focusing on a more comprehensive reference range of SST thickness. A multiple-reference-point procedure for SST thickness might be the most precise and relevant procedure in diagnosing and monitoring morphological alterations in this tendon. Therefore, we aimed to evaluate SST thickness at different levels of the tendon structure localization to identify changes in supraspinatus tendon thickness in specific areas between swimmers with SS tendinopathy and a matched control group. Secondly, we aimed to identify the relative and absolute intra- and inter-rater reliability of STT thickness. Swimmers with SS tendinopathy will have significantly different SST thickness at specific tendon locations compared to a matched control group. Additionally, the ultrasound measurements of SST thickness will demonstrate high relative and absolute intra- and inter-rater reliability when using a procedure with multiple reference points.

## 2. Methods

### 2.1. Study Design

**Figure 1:**
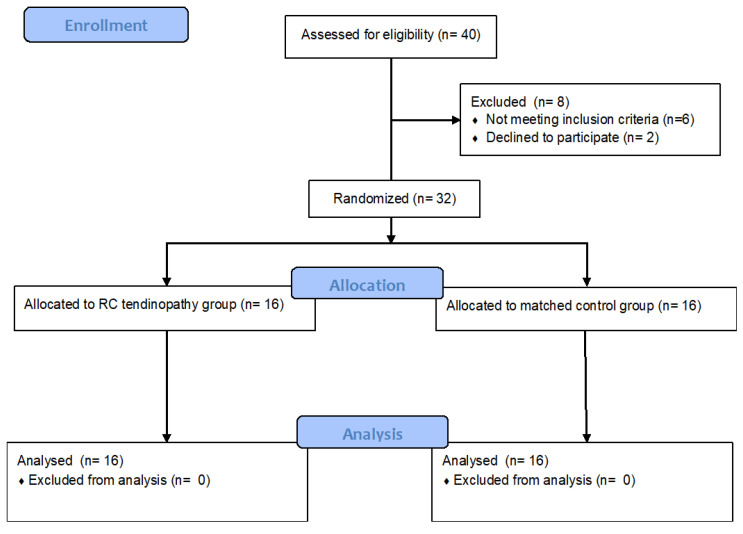
Flowchart diagram of study recruitment.

A cross-sectional observational study with a diagnostic accuracy design was conducted between September 2022 and December 2022 in a biomechanics laboratory. To enhance the presentation quality of this report, the directives for Reporting Reliability and Agreement Studies (GRRAS) [15] were used. The study design was conducted according to the Consolidated Standards of Reporting Trials (CONSORT) for pragmatic trials. During the recruitment process, all participants were asked to avoid strength training and high-intensity and high-volume swimming training for at least 48 h before data collection. During the enrolment, eight participants were excluded from this study, some because they did not meet the inclusion criteria (*n* = 6) and some because they declined to participate in this study (*n* = 2) (Figure 1). All participants read and signed an informed consent form approved by the Senate Research Ethics Committee (project identification code: 1/2019 approval date: 11 January 2019). The study was conducted according to the Declaration of Helsinki.

### 2.2. Participants

The population consisted of thirty-two national-level adolescent swimmers (age: 19.1 ± 1.0 years old; height 175 ± 4.8 cm; weight 75.0 ± 5.1 kg; and BMI 23.5 ± 1.3 kg/m^2^). Participants were distributed into two groups, i.e., swimmers with SS tendinopathy (*n* = 16) and a matched control group of swimmers without SS tendinopathy (*n* = 16). Characteristics, including information on the participants’ training, are presented in Table 1. All swimmers met the following inclusion criteria: (1) training experience ≥6 years and (2) training duration >15 h per week for the past 6 months. The exclusion criteria were as follows: (1) previous injury, i.e., fracture and dislocation of the involved shoulder, (2) previous surgery on the involved shoulder, and (3) inability or unwillingness to participate in exercise tests.

Before the data collection, each participant took part in the recruitment process, including an interview based on basic information about training experience, duration, and training load (per week), as well as a standardized Shoulder Service Questionnaire [16]. This questionnaire contains questions about pain sensations, daily sports activities, work, contentment, and points for development. After, a screening evaluation using Hawkin’s test was performed to provoke a pain sensation in the shoulder during a standing position with the shoulder in abduction (90°) and internal rotation of the forearm. Finally, an ultrasound assessment of the SST was performed by an experienced therapist (eight years of experience) and analyzed by an experienced orthopedic surgeon (fifteen years of experience) to diagnose SS tendinopathy. Both the therapist and surgeon specialized in rotator cuff disorders and injuries. The criteria to recruit swimmers to the RC tendinopathy group were as follows: (1) a positive Hawkin’s test, SST thickening, subacromial–subdeltoid bursa effusion, and hypoechoic areas within the tendon structure.

### 2.3. Sample Size

The sample size was estimated using G*Power software (version 3.1.9.2; Kiel University, Kiel, Germany). To assume differences in SST thickness between swimmers with and without SS tendinopathy, we used a mean of 1.2 mm as the expected mean difference, and a standard deviation of 1.0 mm [6]. The sample size was estimated with the independent samples *t*-test, with an α of 0.05, and a minimum expected effect size (Cohen’s d) of 1.3 was set, with a β of 0.90. The procedure included 14 participants per group. Moreover, to estimate the effect size for a reliability analysis, we used the sample size calculator produced by Arifin [17]. The sample size was estimated for 14 participants per group, and a minimum acceptable reliability (ICC) (ρ0): 0.60, expected reliability (ICC) (ρ1): 0.80, and power of 0.80 were set.

### 2.4. Ultrasound Imaging Acquisition and Measurements

SST thickness was measured using ultrasonography imaging (Alpinion X-CUBE 90, Opinion, Seoul, South Korea) with a linear array transducer (3.0 to 19.0 MHz; 60 mm; SL3-19X; X+ Crystal Signature; Alpinion; Seoul, South Korea) in grey-scale B-mode. Each participant was positioned on a chair with the back supported against a low-back chair. The hand was positioned posteriorly with the palmar side on the superior aspect of the iliac wing, while the elbow was flexed. To obtain SST thickness measurements in the transverse view, the US probe was positioned on the anterior aspect of the shoulder, positioned perpendicular to the SST, and moved to the anterior–lateral margin of the acromion [6]. The images were obtained by the same examiner (SK) with eight years of experience in ultrasound imaging, while two raters (SK and MGP) performed the SST thickness measurements on the captured images. Both raters were certified physical therapists with over five years of experience (five and seven years, respectively) in musculoskeletal US imaging. Before data collection, the examiner underwent a 2 h training session on ultrasound machine operation, imaging settings, and examination procedures, including participant positioning and US probe usage. The examiner acquired two SST images within 10 min and repeated the same procedure after 30 min. Measurements were independently obtained by both examiners in a randomly selected order after a 1-week interval. Additionally, SST images were stored for a month after data collection. Both examiners were blinded to the coded images, which were later decoded by the principal investigator. The average of each measurement from each of the two images at the test and retest sessions was used for statistical analyses.

**Figure 2:**
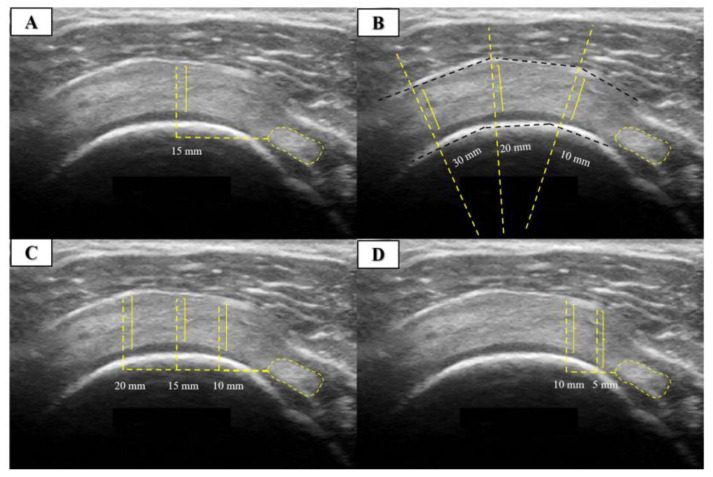
Ultrasound measurement of the supraspinatus tendon thickness in short axis. (**A**) Measurements were taken at 15 mm [5], (**B**) at 10, 20 and 30 mm [13], (**C**) at 10, 15 and 20 mm [6], and (**D**) at 5 and 10 mm [4] lateral to the most hyperechogenic reference point of the biceps tendon.

The measurement procedure for SST thickness consisted of four different measurement procedures performed and published over the last decade. The first measurement procedure took measurements at 15 mm [5], the second at 10, 20, and 30 mm [13], the third at 10, 15, and 20 mm [6], and the fourth at 5 and 10 mm [4] lateral to the most hyperechogenic reference point of the biceps tendon. All measurement procedures contained more than a single reference point that was averaged. An illustrative example of US images is shown in Figure 2.

### 2.5. Statistical Analysis

Statistical analysis was performed using the statistical software SPSS 21 (SPSS Inc., Chicago, IL, USA). Descriptive statistics (mean ± standard deviation (SD) were calculated. Normality was evaluated using the Shapiro–Wilk normality test. To determine differences between swimmers with SS tendinopathy and the control group, an independent samples *t*-test was used. Further, a two-way analysis of variance (ANOVA) with the procedure (*n*. 1-2-3-4) and group (tendinopathy or controls) was also performed. If an interaction between variables was found, the Bonferroni test was used as a post hoc test (*p* = 0.01). The effect size was estimated using partial eta square (η^2^) and classified as small (0.2< η^2^ < 0.49), medium (0.5< η^2^ < 0.79), or large (η^2^ ≤ 0.8). A *p*-value <0.05 was considered statistically significant. Finally, we estimated intra- and inter-rater reliability as a two-way random, absolute agreement, and single measures model (ICC2.3). Reliability was classified as poor (ICC < 0.5), moderate (0.5 < ICC < 0.69), good (0.7 < ICC < 0.89), or excellent (ICC > 0.9) [18]. The standard error of measurement (SEM = SD·√(1 − ICC) and the minimal detectable change (MDC = SEM·1.96·√2) were also calculated [19].

## 3. Results

Table 2 shows the mean and SD values of SST thickness in the four measurement procedures in swimmers with and without tendinopathy. The independent samples t-test showed significantly greater SST thickness in swimmers with SS tendinopathy than in those without tendinopathy for each procedure (all, *p* < 0.001, Table 1). The two-way ANOVA revealed a statistically significant main effect for the procedure (F_3, 240_ = 6.9, *p* < 0.001, η^2^ = 0.08) and group (F_1, 240_ = 1598.6, *p* < 0.001, η^2^ = 0.87) without a significant group and procedure interaction effect. Mean SST thickness was greater within procedure no. 1 than in procedure no. 3 in both groups (*p* < 0.001).

Intra-rater reliability ranged from good to excellent (ICC_2.3_ from 0.78 to 0.99) in both groups. The ICCs for rater 1 were excellent for all measure procedures among swimmers with SS tendinopathy (from 0.91, 95%CI 0.60–0.93 to 0.98, 95%CI 0.95–0.99) and controls (from 0.91, 95%CI 0.21 to 0.93 to 0.99, 95%CI 0.96 to 0.99). The intra-rater ICCs for rater 1 were higher than those obtained by rater 2 in both groups. The ICCs of rater 2 ranged from 0.82 (95% CI 0.33–0.85) to 0.96 (95%CI 0.93–0.99) for swimmers with SS tendinopathy and from 0.78 (95%CI 0.23–0.84) to 0.99 (95%CI 0.96–0.99) for the control group. Inter-rater reliability was good and excellent among the SS tendinopathy group (from 0.83, 95%CI 0.36–0.89 to 0.97, 95% CI 0.84–0.98) and control group (from 0.87, 95%CI 0.47–0.91 to 0.96, 95%CI 0.76–0.97) (Table 3).

The absolute reliability was defined by SEM and MDC calculations. For rater 1, the intra-rater SEMs ranged from 0.08 to 0.13 mm in swimmers with SST tendinopathy and from 0.04 mm to 0.16 mm in the control group. Moreover, SEMs were higher for procedures 1 and 3 in swimmers with SST tendinopathy and lower in procedures 2 and 4, than within controls. However, for rater 2, the SEMs ranged from 0.10 mm to 0.20 mm in swimmers with SST tendinopathy and from 0.06 mm to 0.19 mm in controls. The inter-rater SEMs ranged from 0.05 mm to 0.19 mm in swimmers with SST tendinopathy and from 0.04 mm to 0.18 mm in controls (Table 3).

Finally, the intra-rater MDC ranged from 0.21 mm to 0.36 mm in swimmers with SS tendinopathy, from 0.12 mm to 0.43 mm in the matched control group for rater 1, and from 0.28 mm to 0.57 mm in swimmers with SS tendinopathy and from 0.16 mm to 0.53 mm in the control group for rater 2. The inter-rater MDC ranged from 0.14 mm to 0.54 mm in swimmers with SS tendinopathy and from 0.12 mm to 0.45 mm in controls (Table 3).

## 4. Discussion

This study is the first to report reliability for the metrology of SST thickness in subjects with and without tendinopathy using single-, double-, and multi-location reference points. Our results found significantly greater SST thickness in swimmers with SS tendinopathy when compared with matched control swimmers. Moreover, our study showed that SST thickness measurements are a reliable method by reporting relative (expressed by ICCs) intra- and inter-rater reliability as good to excellent for four different measurement procedures. Finally, the absolute reliability (expressed by SEM and MDC) also showed higher values in procedures no. 2 and 3 (multi-location measurements). Our study demonstrated that procedure no. 3 was the most reliable location with the lowest error rate. Current findings might help to understand the importance of using a precise methodology to define SST tendinopathy and other chronic alterations in this tendon.

We observed significantly greater SST thickness in swimmers with tendinopathy than in those without tendinopathy. A percentage difference between both groups of 24% (rater 1) and 28% (rater 2) was identified. By averaging the results for both raters, we noticed that the lowest difference between swimmers with SS tendinopathy and controls was found for the single-location measurement, i.e., procedure no. 1 (25%), while in other procedures (double—procedure no. 4; multi-measurement locations—procedures no. 2 and 3) the difference ranged between 27% and 28%. In agreement with our results, McCreesh [4] also reported greater SST thickness in swimmers with rotator cuff tendinopathy (13%). According to previous studies, greater SST thickness might be related to some intrinsic mechanisms, i.e., increased blood flow, increased water content, and acute inflammation [4,6,20]. This observation may suggest that measurement procedures based on multiple reference points might be the most valid procedures to investigate morphological alterations in the SST.

Our analysis reported good to excellent intra- (ICCs ranged from 0.78 to 0.99) and inter-rater reliability (ICCs ranged from 0.83 to 0.97) for the procedures. More specifically, we found the lowest intra-rater reliability for procedure no. 2 (ICCs ranging from 0.78 to 0.91 for the SS tendinopathy group, and from 0.83 to 0.87 for the control group) while the highest was found for procedure no. 1 (ICCs of 0.98 and 0.96 for SS tendinopathy group) and procedure no. 3 (both 0.99 for the control group). The lowest inter-rater reliability was seen for procedure no. 2 (SS tendinopathy group) and procedure no. 4 (control group) (both ICCs: 0.83), while the highest was found for procedure no. 3 (0.97 and 0.96 for both groups). The relative reliability of SST thickness has been reported as good to excellent for intra- (ICCs from 0.88 to 0.97) and inter-rater (ICCs from 0.94 to 0.98) reliability in previous studies [4,6,13,21]. These ICCs were based on different procedures, including single- [21], double- [4], and triple-measurement locations [6,13]. Our relative intra- and inter-rater reliability differs from those obtained previously in these papers; however, it should be noted that only McCreesh et al. [4] and Ahmad et al. [21] aimed to investigate the reliability of SST thickness. In contrast, the remaining studies used only intra- or inter-rater reliability to description accuracy [6,13]. Therefore, we searched for studies that included reliability in their analysis of one of the measurement procedures of the SST. The PubMed database showed the greatest number of studies (*n* = 6) citing procedure no. 3, investigated by Michener et al. [6]. The other procedures were cited once—procedure no. 2 [21]; twice—procedure no. 4 [22,23]; and three times—procedure no. 1 [11,21,24]. For procedure no. 1, the intra-rater reliability was excellent, with ICCs between 0.98 and 0.99, and inter-rater reliability had ICCs of 0.95 (for both studies). The lowest intra-rater reliability was found in a study performed by Leong et al. [13], where the ICC was 0.83. In procedure no. 4, the intra-rater reliability was excellent (ICC 0.91), while inter-rater reliability was medium (ICC 0.64) [23]. For procedure no. 3, the intra-rater reliability was excellent, with ICCs ranging from 0.91 to 0.99 [10,12,25,26,27,28]. It should be noted that in four studies, the ICC was 0.98 or higher [12,25,26,27,28]. The inter-rater reliability was excellent, with ICCs ranging from 0.90 to 0.94 [10,12]. The results from our study have shown that procedure no. 2 had the lowest reliability, while procedures no. 1 and 3 had the highest reliability. In particular, single and multiple reference points are reliable, and both Leong et al. [13] and Michener et al. [6] included multiple reference point analysis. From our perspective, this procedure (no. 2) has some limitations based on the methodology of measurement making it more difficult to perform. These findings might be similar to Tsui et al. [20], who also identified lower ICCs; however, these authors still established good reliability.

The absolute reliability of SST thickness assessment, expressed by SEM and MDC, has been reported in previous papers as well. An analysis showed that fewer studies calculated both SEM and MDC for their analysis of reliability. In our study, we reported low absolute intra-rater reliability for all procedures except no. 2. The SEMs ranged from 0.04 mm to 0.18 mm, while the MDC ranged from 0.12 mm to 0.42 mm. However, the lowest value was found for procedure no. 3, with SEMs ranging from 0.04 mm to 0.10 mm and MDCs from 0.12 mm to 0.28 mm. For procedure no. 2, the SEM ranged from 0.13 mm to 0.20 mm, while the MDC ranged from 0.36 mm to 0.57 mm. For inter-rater reliability, we found the lowest SEM and MDC for procedure no. 3 (swimmers with SS tendinopathy, SEM: 0.05 mm; MDC: 0.10 mm) and procedure no. 1 (controls, SEM: 0.04 mm; MDC: 0.12 mm). Only McCreesh et al. [4] reported the SEM and MDC, while Leong et al. [13] reported the SEM and Michener et al. [6] only the MDC. Leong et al. [13] reported a SEM of 0.23 mm (intra-rater reliability), whereas Michener et al. [6] reported an MDC of 0.40 mm. McCreesh et al. [4] showed SEMs of 0.20 mm and 0.30 mm (intra-rater reliability), 0.50 mm (inter-rater reliability), and also MDCs of 0.06 mm (intra-rater reliability) and 1.3 mm (inter-rater reliability). Previous studies have shown SEMs ranging from 0.05 mm to 0.33 mm and an MDC of 0.91 [11,21,24] for procedure no. 1; SEMs from 0.05 mm to 0.40 mm and MDCs from 0.10 mm to 0.50 mm [10,12,25,26,27,28] for procedure no. 3; and an MDC of 0.60 mm [22] for procedure no.4.

Rotator cuff ultrasonography is usually used and recommended to evaluate morphological properties, such as SST thickness, in people with tendinopathies, tears, and other injuries [6]. Analysis of SST thickness in swimmers may provide crucial information and clinical outcomes about alterations in morphological properties. Clinically, our findings indicate that ultrasound measurement procedures at multiple points might be successfully used in the early diagnosis, monitoring, and management of SS tendinopathy in patients and overhead athletes. Moreover, the approach should focus on a simplified measurement model that involves a maximal distance of 20 mm from the reference point on the biceps tendon. The research findings suggest that the most reliable measurement procedure, as presented by Michener et al. [6], should be used as the “gold standard” for SST injuries during individual diagnosis and treatment interventions to prevent progression and improve patient outcomes. Regular ultrasound evaluation of the SST can be integrated into physician and physical therapy routines for screening and injury prediction in athletes, especially in the detection of subclinical impingement syndrome, tendinopathies, and tears. On the other hand, these findings could support the development of a tendon degeneration model. The mechanisms related to greater SST thickness in tendinopathy might be due to increased cellularity and alterations in collagen fiber disorganization [29]. Moreover, a common symptom of tendinopathy in the rotator cuff structures is increased fluid content in soft tissues and inflammation [30,31]. SS tendinopathy is caused by greater training intensity and volume in swimmers, leading to shoulder pain and dysfunction [26]. Finally, it should be also noted that the mechanisms of rotator cuff tendinopathy are not explained well enough; therefore, ultrasonographical studies should be performed to analyze morphological properties [25].

Some limitations should be pointed out to improve future studies. First, we recruited adolescent swimmers with an average age of 19 years. Future studies should evaluate SST thickness in adults and swimmers with greater training experience. Moreover, future studies could analyze differences between swimming strokes and relation to training intensity and volume in different periods of macrocycles [31]. Second, future studies should investigate sex-related differences in SST thickness. Finally, we used four different measurement procedures with single and multiple reference points; however, future studies could focus on more distal points.

## 5. Conclusions

Our study evaluated SST thickness and identified relative and absolute reliability in swimmers with and without SS tendinopathy in adolescent swimmers. The SST was thicker among swimmers with SS tendinopathy than in those without tendinopathy. Further, thickness was also greater in the middle portion of the tendon measured at 10 mm from the biceps tendon. This may suggest a heterogeneous distribution of thickness of the SST. Ultrasound imaging is a reliable method to investigate SST thickness using different measurement procedures. A multiple-reference-point procedure including a simple methodology (10, 15, and 20 mm from biceps tendon) was defined as the most reliable, expressed by the highest intra- and inter-rater ICCs and the lowest SEMs and MDCs. Procedures should include this multiple-measurement approach along the entire visible portion of the SST. 

## Figures and Tables

**Table 1:** Mean ± SD of the participant characteristics in rotator cuff (RC) tendinopathy and matched control group.

Variables	RC Tendinopathy Group	Matched Control Group
Age (year)	19.0 ± 0.8	19.1 ± 1.0
Gender	♂: 8; ♀:8	♂: 9; ♀:7
Body height (m)	1.76 ± 3.5	1.75 ± 2.6
Body mass (kg)	75.2 ± 5.7	74.2 ± 7.3
Body Mass Index (kg/m^2^)	24.2 ± 0.5	24.7 ± 0.9
Rotator cuff tendinopathy:		
Right	16	N/A
Left	0	N/A
Training experience (years)	8 ± 1	7 ± 1
Training duration (hour/week)	16 ± 1	16 ± 1

N/A—not applicable.

**Table 2:** Ultrasound measurements for SST thickness (mm) in four different measurement procedures in swimmers with rotator cuff (RC) tendinopathy and matched control group.

		RC Tendinopathy Group (*n* = 16)	Matched Control Group (*n* = 16)	*p*-Values
Procedure no. 1	Rater 1	6.31 ± 0.5	4.69 ± 0.1	*p* < 0.001
	Rater 2	6.22 ± 0.6	4.72 ± 0.1	*p* < 0.001
Procedure no. 2	Rater 1	6.19 ± 0.3	4.49 ± 0.3	*p* < 0.001
	Rater 2	6.15 ± 0.4	4.45 ± 0.3	*p* < 0.001
Procedure no. 3	Rater 1	6.15 ± 0.3	4.44 ± 0.3	*p* < 0.001
	Rater 2	6.20 ± 0.2	4.46 ± 0.4	*p* < 0.001
Procedure no. 4	Rater 1	6.09 ± 0.2	4.39 ± 0.1	*p* < 0.001
	Rater 2	5.96 ± 0.3	4.43 ± 0.1	*p* < 0.001

**Table 3:** Intra-class correlation coefficients (ICCs), 95% confidence interval (95% CI), standard error of measurement (SEM), minimal detectable change (MDC), and 95% limit of agreement (LOA) for intra- and inter-rater reliability of SST thickness.

		RC Tendinopathy Group (*n* = 16)				Matched Control Group (*n* = 16)			
		Procedure no. 1	Procedure no. 2	Procedure no. 3	Procedure no. 4	Procedure no. 1	Procedure no. 2	Procedure no. 3	Procedure no. 4
Intra-rater									
Rater 1	ICC	0.98	0.91	0.93	0.91	0.94	0.91	0.99	0.93
	95% CI	0.95–0.99	0.60–0.93	0.66–0.95	0.70–0.93	0.72–0.96	0.21–0.93	0.96–0.99	0.66–0.95
	SEM [mm]	0.09	0.13	0.08	0.08	0.05	0.16	0.04	0.13
	MDC [mm]	0.26	0.36	0.23	0.21	0.14	0.43	0.12	0.35
	LOA	−0.22; 0.38	−0.38; 0.36	−0.10; 0.50	−0.19; 0.20	−0.14; 0.10	−0.18; 0.56	−0.03; 0.04	−0.20–0.60
Rater 2	ICC	0.96	0.82	0.92	0.90	0.92	0.78	0.99	0.89
	95% CI	0.93–0.99	0.33–0.85	0.60–0.93	0.53–0.92	0.59–0.94	0.23–0.84	0.96–0.99	0.36–0.91
	SEM [mm]	0.13	0.20	0.10	0.12	0.06	0.19	0.09	0.18
	MDC [mm]	0.34	0.57	0.28	0.33	0.16	0.53	0.20	0.42
	LOA	−0.18; 0.03	−0.05; 1.05	−0.79; 0.64	−0.53–0.97	−0.16; 0.10	−0.82; 0.73	−0.08; 0.02	−0.86–1.00
Inter-rater	ICC	0.94	0.83	0.97	0.85	0.96	0.87	0.96	0.83
	95% CI	0.68–0.96	0.36–0.89	0.84–0.98	0.31–0.89	0.76–0.97	0.47–0.91	0.81–0.97	0.33–0.88
	SEM [mm]	0.18	0.19	0.05	0.10	0.04	0.14	0.08	0.18
	MDC [mm]	0.50	0.54	0.14	0.29	0.12	0.40	0.22	0.45
	LOA	−0.20; 0.50	−0.50; 0.53	−0.68; 0.38	−0.42; 0.67	−0.12; 0.05	−0.37; 0.40	−0.43; 0.77	−0.49; 0.49

## Data Availability

The authors confirm that the data supporting the findings of this study are available within the article.

## References

[1] Sein M.L., Walton J., Linklater J., Appleyard R., Kirkbride B., Kuah D., Murrell G.A. (2010). Shoulder pain in elite swimmers: Primarily due to swim-volume-induced supraspinatus tendinopathy. Br. J. Sports Med..

[2] Kositsky A., Gonçalves B.A.M., Stenroth L., Barrett R.S., Diamond L.E., Saxby D.J. (2020). Reliability and Validity of Ultrasonography for Measurement of Hamstring Muscle and Tendon Cross-Sectional Area. Ultrasound. Med. Biol..

[3] Neer C.S. (1972). Anterior acromioplasty for the chronic impingement syndrome in the shoulder: A preliminary report. J. Bone Joint Surg. Am..

[4] McCreesh K.M., Anjum S., Crotty J.M., Lewis J.S. (2016). Ultrasound measures of supraspinatus tendon thickness and acromiohumeral distance in rotator cuff tendinopathy are reliable. J. Clin. Ultrasoud..

[5] Cholewinski J.J., Kusz D.J., Wojciechowski P., Cielinski L.S., Zoladz M.P. (2008). Ultrasound measurement of rotator cuff thickness and acromio-humeral distance in the diagnosis of subacromial impingement syndrome of the shoulder. Knee Surg. Sports Traumatol. Arthrosc..

[6] Michener L.A., Subasi Yesilyaprak S.S., Seitz A.L., Timmons M.K., Walsworth M.K. (2015). Supraspinatus tendon and subacromial space parameters measured on ultrasonographic imaging in subacromial impingement syndrome. Knee Surg. Sports Traumatol. Arthrosc..

[7] Porter K.N., Talpey S., Pascoe D., Blanch P.D., Walker H.M., Shield A.J. (2021). The effect of swimming volume and intensity on changes in supraspinatus tendon thickness. Phys. Ther. Sport.

[8] Dischler J.D., Baumer T.G., Finkelstein E., Siegal D.S., Bey M.J. (2018). Association between years of competition and shoulder function in collegiate swimmers. Sports Health.

[9] So B.C.L., Lau S.C.T., Kwokm W.Y., Tse D.H.T., Man S.S. (2023). Investigating the association between supraspinatus tendon abnormality, shoulder pain and isokinetic strength in elite swimmers: A cross-sectional study. J. Sports Sci. Med..

[10] BaĞcier F., KÜlcÜ G.D., Yorulmaz E., Altunok E.Ç. (2020). Intra- and inter-rater reliability of ultrasound measurements of supraspinatus tendon thickness, acromiohumeral distance, and occupation ratio in patients with shoulder impingement syndrome. Arch. Rheumatol..

[11] Hunter D.J., Rivett D.A., McKiernan S., Snodgrass S.J. (2021). Acromiohumeral distance and supraspinatus tendon thickness in people with shoulder impingement syndrome compared to asymptomatic age and gender-matched participants: A case control study. BMC Musculoskelet. Disord..

[12] Porter K.N., Blanch P.D., Walker H.M., Shield A.J. (2020). The effect of previous shoulder pain on supraspinatus tendon thickness changes following swimming practice. Scand J. Med. Sci. Sports.

[13] Leong H.-T., Tsui S., Ying M., Leung V.Y., Fu S.N. (2012). Ultrasound measurements on acromio-humeral distance and supraspinatus tendon thickness: Test–retest reliability and correlations with shoulder rotational strengths. J. Sci. Med. Sport.

[14] Kretić D., Turk T., Rotim T., Šarić G. (2018). Reliability of Ultrasound Measurement of Muscle Thickness in Patients with Supraspinatus Tendon Pathology. Acta Clin. Croat..

[15] Kottner J., Audigé L., Brorson S., Donner A., Gajeweski B.J., Hróbjartsson A., Robersts C., Shoukri M., Streiner D.L. (2011). Guidelines for Reporting Reliability and Agreement Studies (GRRAS) were proposed. J. Clin. Epidemiol..

[16] L’insalata J.C., Warren R.F., Cohen S.B., Altchek D.W., Peterson M.G. (1997). A self-administered questionnaire for assessment of symptoms and function of the shoulder. J. Bone Joint Surg. Am..

[17] Arifin W.N. (2018). A Web-based Sample Size Calculator for Reliability Studies. Educat. Med. J..

[18] Koo T.K., Li M.Y. (2016). A Guideline of Selecting and Reporting Intraclass Correlation Coefficients for Reliability Research. J. Chiropr. Med..

[19] Weir J.P. (2005). Quantifying test-retest reliability using he intraclass correlation coefficient and the SEM. J. Strength Cond. Res..

[20] Tsui S.S., Leong H.T., Leung V.Y., Ying M., Fu S.N. (2017). Tendon vascularity in overhead athletes with subacromial pain syndrome and its correlation with the resting subacromial space. J. Shoulder Elbow. Surg..

[21] Ahmad A., Bandpei M.A.M., Gilani S.A., Munawar A., Ahmed I., Tanveer F. (2017). Reliability of musculoskeletal ultrasound imaging to measure supraspinatus tendon thickness in healthy subjects. J. Phys. Ther. Sci..

[22] Gala-Alarcón P., Prieto-Gómez V., Bailón-Cerezo J., Yuste-Sánchez M.J., Arranz-Martín B., Torres-Lacomba M. (2021). Changes in shoulder outcomes using ultrasonographic assessment of breast cancer survivors: A prospective longitudinal study with 6-month follow-up. Sci. Rep..

[23] Yuan X., Lowder R., Aviles-Wetherell K., Skroce C., Yao K.V., Soo Hoo J. (2022). Reliability of point-of-care shoulder ultrasound measurements for subacromial impingement in asymptomatic participants. Front. Rehabil. Sci..

[24] Elgyoum A.M., Mohammed M.H., Abdelrahim A., Zidan M.M., Fagiry M.A., Salih M., Elhaj M., Davidson R., Mahmoud M.Z. (2021). Measurements of rotator cuff tendons, acromioclavicular joint space, and subacromion-subdeltoid bursa in the adults sudanese population using ultrasonography. J. Radiat. Res. Appl. Sci..

[25] Klich S., Madeleine P., Ficek K., Sommer K., Fernández-de-Las-Peñas C., Michener L.A., Kawczyński A. (2023). Functional and morphological changes in shoulder girdle muscles after repeated climbing exercise. Res. Sports Med..

[26] Klich S., Pietraszewski B., Zago M., Galli M., Lovecchio N., Kawczyński A. (2020). Ultrasonographic and Myotonometric Evaluation of the Shoulder Girdle After an Isokinetic Muscle Fatigue Protocol. J. Sport Rehabil..

[27] Klich S., Kisilewicz A., Pożarowszczyk B., Fic M., Seidel W., Chromik K., Kawczyński A. (2017). Reliability of ultrasound measures of supraspinatus tendon thickness and subacromial space in judo athletes. Arch. Budo Sci. Martial Arts.

[28] Klich S., Kisilewicz A., Pożarowszczyk B., Zatoń M., Kawczyński A., Michener L.A. (2019). Shoulder tendon characteristics in disabled swimmers in high functional classes—Preliminary report. Phys. Ther. Sports.

[29] Neviaser A., Andarawis-Puri N., Flatow E. (2012). Basic mechanisms of tendon fatigue damage. J. Shoulder Elbow. Surg..

[30] Gimbel J.A., Van Kleunen J.P., Mehta S., Perry S.M., Williams G.R., Soslowsky L.J. (2004). Supraspinatus tendon organizational and mechanical properties in a chronic rotator cuff tear animal model. J. Biomech..

[31] Matzkin E., Suslavich K., Wes D. (2016). Swimmer’s Shoulder: Painful Shoulder in the Competitive Swimmer. J. Am. Acad Orthop. Surg..

